# Use of Copper to Selectively Inhibit *Brachionus calyciflorus* (Predator) Growth in *Chlorella kessleri* (Prey) Mass Cultures for Algae Biodiesel Production

**DOI:** 10.3390/ijms160920674

**Published:** 2015-08-31

**Authors:** Vishnupriya Pradeep, Steven W. Van Ginkel, Sichoon Park, Thomas Igou, Christine Yi, Hao Fu, Rachel Johnston, Terry Snell, Yongsheng Chen

**Affiliations:** 1 School of Civil and Environmental Engineering, Georgia Institute of Technology, 200 Bobby Dodd Way, Atlanta, GA 30313, USA; E-Mails: priya71189@gmail.com (V.P.); svg7@mail.gatech.edu (S.W.V.G.); sichoon.park@gmail.com (S.P.); thomas.igou@gmail.com (T.I.); c.yi@gatech.edu (C.Y.); hfu36@gatech.edu (H.F.); 2 School of Biology, Georgia Institute of Technology, 310 Ferst Drive, Atlanta, GA 30313, USA; E-Mails: rachel.johnston@biology.gatech.edu (R.J.); terry.snell@biology.gatech.edu (T.S.)

**Keywords:** biodiesel production, algae pond crash, toxicity, rotifer, copper

## Abstract

A single *Brachionus* rotifer can consume thousands of algae cells per hour causing an algae pond to crash within days of infection. Thus, there is a great need to reduce rotifers in order for algal biofuel production to become reality. Copper can selectively inhibit rotifers in algae ponds, thereby protecting the algae crop. Differential toxicity tests were conducted to compare the copper sensitivity of a model rotifer—*B. calyciflorus* and an alga, *C. kessleri*. The rotifer LC_50_ was <0.1 ppm while the alga was not affected up to 5 ppm Cu(II). The low pH of the rotifer stomach may make it more sensitive to copper. However, when these cultures were combined, a copper concentration of 1.5 ppm was needed to inhibit the rotifer as the alga bound the copper, decreasing its bioavailability. Copper (X ppm) had no effect on downstream fatty acid methyl ester extraction.

## 1. Introduction

Biofuels are an attractive source of sustainable energy for the future due to a reduction in carbon emissions and the ability to supplement petroleum based transportation fuels. Algal biofuel has higher areal productivity and lipid content than terrestrial biofuel crops [1]. However, like terrestrial crops, algae are susceptible to predation by higher organisms including rotifers, protozoa, and ciliates [2].

**Figure 1:**
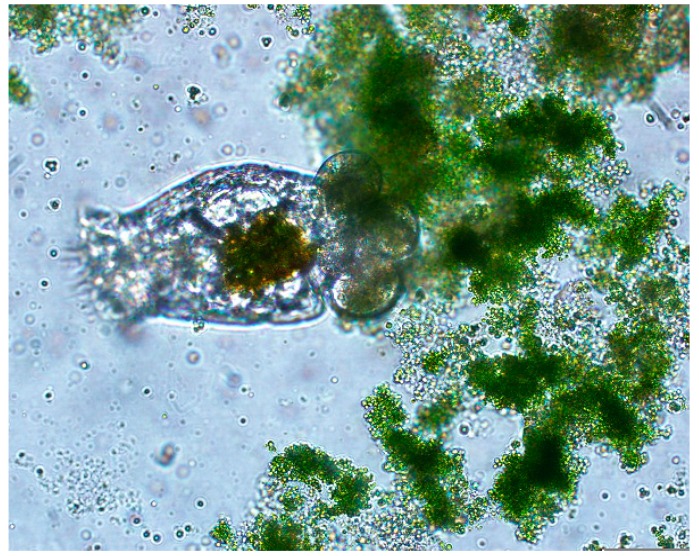
*B. calyciflorus* carrying three eggs and feeding on *C. kessleri* cells (taken at 40× magnification using an Olympus IX51 microscope).

Figure 1 shows the rotifer, *Brachionus calyciflorus*, feeding on *Chlorella kessleri* algal cells. Studies have shown that a single rotifer can consume up to 115,000 *Nanochloropsis* sp. cells per day which will lead to an algae “pond crash” [2]. Rotifers provide a service to an aquatic community as they are top tier algae predators able to completely digest algae with a crushing jaw and a stomach. Rotifer manure supports the growth of bacteria, ciliates, and other protists that feed on bacteria. Other algae predators don’t have a true jaw and must engulf algae cells making their consumption rate much slower [3]. As part of the Algae Testbed Public Private Partnership (ATP^3^), rotifers and several other predators (Vorticella, ciliates, amoebas, golden algae, *etc*.) were observed in freshwater mass cultures of *C. vulgaris* which limited production to just a few weeks.

Rotifers are known to be sensitive to a wide range of chemicals including sodium hypochlorite, mercury, cadmium, zinc, aluminum, and copper [4]. The concentration of a chemical lethal to 50% of a test population during a specific time period is known as the LC_50_. The copper 24-h LC_50_ of the three rotifer species, *B. calyciflorus*, *B. rubens*, and *B. plicatilis* are reported to be 0.026, 0.019, and 0.120 ppm [4,5,6,7]. Copper toxicity, biosorption, and the influence of pH on copper toxicity have also been reported for *Chlorella* sp. with the intent to prevent algal blooms in aquaculture [8]. Algae require trace amounts of copper for growth, however excess copper can inhibit photosynthesis and oxidative phosphorylation in the electron transport chain [9]. Copper sulfate is commonly used to control algae in swimming pools at recommended concentrations of 1–2 ppm. According to Ansari and Amjad (2008), maximum *C. vulgaris* growth was observed at 5 ppm Cu(II) and decreased at higher copper concentrations [10].

*C. vulgaris* is reportedly more tolerant to copper than *B. calyciflorus*. Thus, our research study is based on the hypothesis that small amounts of copper can be added to algae ponds to inhibit rotifers without significantly inhibiting the alga. This study bridges a gap in continuous algae cultivation research as no toxicity study has been done specifically to prevent a pond crash by rotifers. It is the hope of this research, that chemical agents, such as copper, can be used in the algal biofuel industry to control predation. Although not part of this study, it is assumed that the copper can be recycled. A first step in biodiesel extraction is acid hydrolysis which can make copper completely soluble in water, enabling recovery.

## 2. Results and Discussion

### 2.1. Effect of Copper on B. calyciflorus and C. kessleri

**Figure 2:**
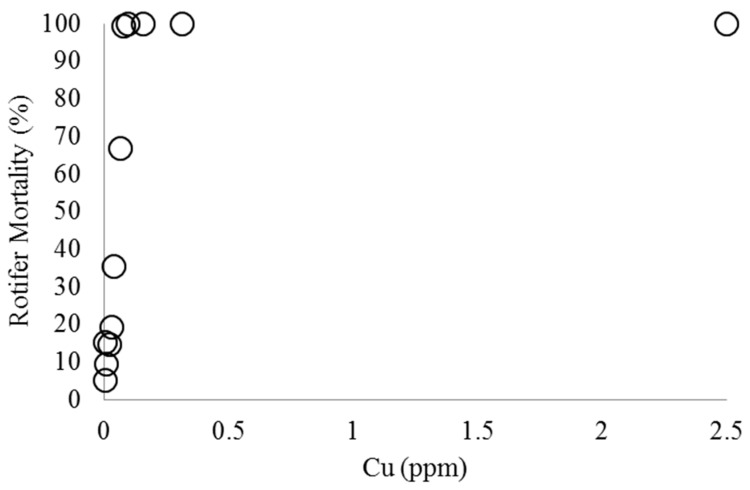
*B. calyciflorus* mortality as a function of copper concentration (Circles represent rotifer mortality at different copper concentrations).

The 24-h copper LC_50_ for *B. calyciflorus* in spring water was determined to be 0.046 ppm Cu(II) with a standard error of 3.09 and upper (53.6) and lower (39.0) 95% confidence limits (*p* < 0.001) (Figure 2). The mortality of the controls was <5% with a relative standard error of 12% (*n* = 31). The rotifer copper LC_50_ in Bold’s Basal Medium (BBM) was found to be 0.041 ppm, which is not significantly different than the value of 0.026 ppm observed by Snell and Persoone [6]. The mortality of the controls was <9% with a relative standard error of 9% (*n* = 4). The small difference in copper toxicity in the spring water compared to BBM is likely due to the low pH of the rotifer stomach. In BBM, the copper is likely bound as an EDTA-Cu complex and is non-toxic to the algae, but since rotifers are filter feeders, the complex dissociates in the rotifer stomach and copper can exhibit its toxic effect.

**Figure 3:**
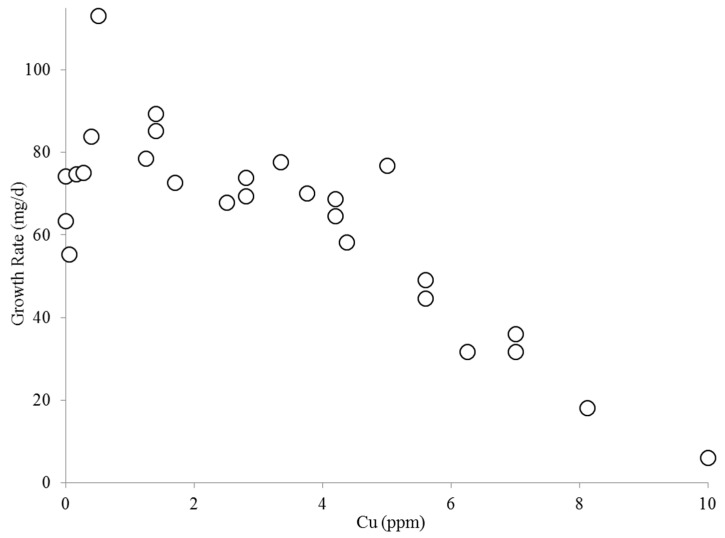
*C. kessleri* growth in 1 L columns as a function of copper concentration (*n* = 27). (Circles represent algae growth rates at different copper concentrations).

**Figure 4:**
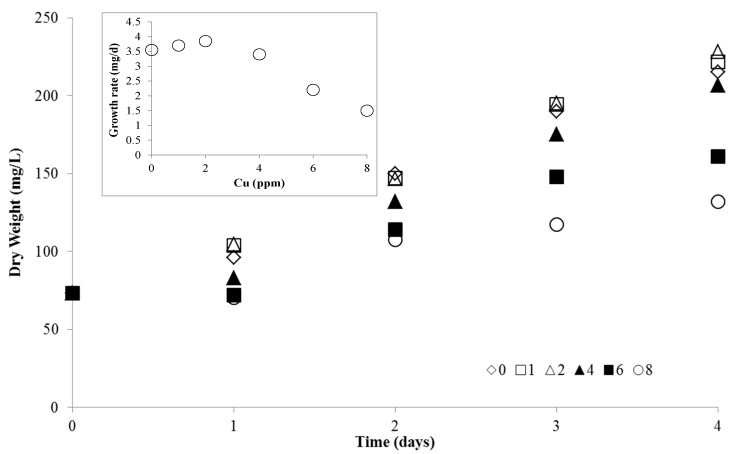
*C. kessleri* growth in 100 mL shake flasks as a function of time at different copper concentrations (ppm). The inset shows the growth rate as a function of the copper concentration.

Before the algal shake flask experiments were conducted, the effect of copper on algae was investigated in 1 L columns in BBM as shown in Figure 3. Similar to the findings reported in [9], *C. kessleri* growth was unaffected up to 5 ppm Cu(II). The algae growth was constant at ~75 mg/day from 0 to 5 ppm Cu(II). Figure 4 shows algal growth in the shake flasks. The algal growth rate was unaffected up to 4 ppm Cu(II) and decreased at higher concentrations (Figure 4 inset). Based on these results, *C. kessleri* is more tolerant to copper than *B. calyciflorus*. In these tests, using 4-day old algae cells, approximately 77% of the copper existed as free copper which shows that rotifers don’t need to ingest algae to ingest copper. In older cultures or crashed cultures, the copper is likely bound to rotifer feces/digested algae or the soluble algal products (SAPs) that algae produce.

### 2.2. C. kessleri Pond Crash Rate

**Figure 5:**
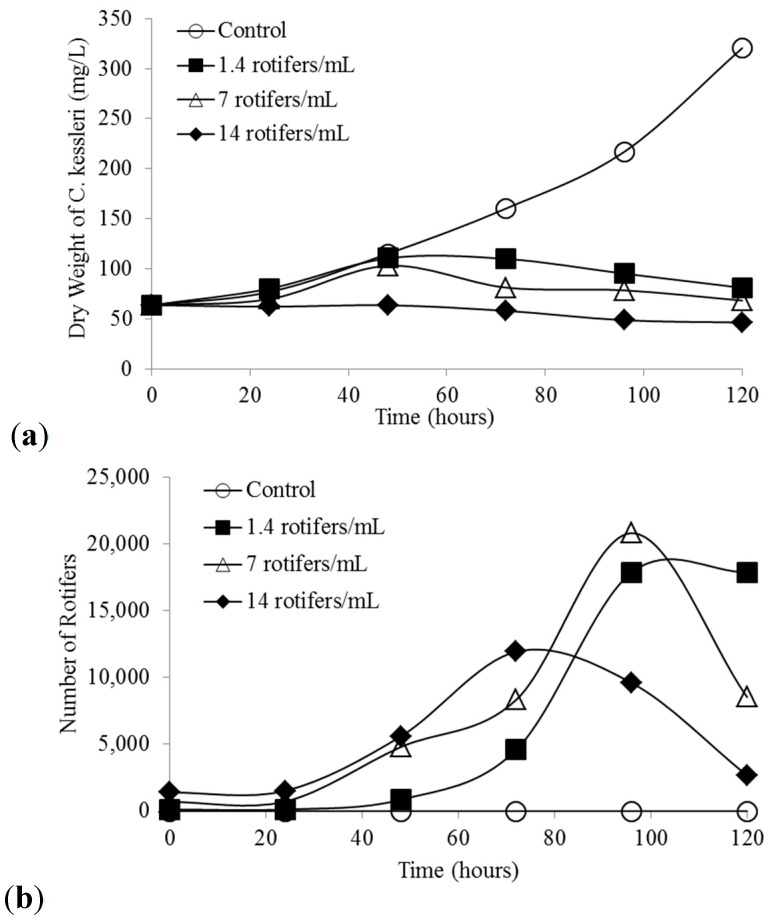
(**a**) *C. kessleri* pond crash rate at three different initial *B. calyciflorus* densities; (**b**) Increase in *B. calyciflorus* during the *C. kessleri* pond crash.

Figure 5a,b shows the co-culture of *C. kessleri* and *B. calyciflorus*. Every instance when rotifers were added, a pond crash resulted. Due to predation, algal dry weight increased with decreasing initial rotifer concentration. As shown in Figure 5b, there was a lag in rotifer growth as it takes newly hatched females about 1 day to initiate reproduction. Rotifer populations grew rapidly as females can produce 20 eggs in their 7 day life span at 25 °C.

Rotifer numbers peaked due to the limited amount of algae for food. The time to peak rotifer concentration decreased with increasing initial rotifer concentration (Figure 5b). The experiment with the highest initial rotifer concentration of 14 rotifers/mL had the lowest peak concentration likely due to the limited amount of time for algae growth (Figure 5b). Based on the data in Figure 5a,b, the doubling times of *C. kessleri* and *B. calyciflorus* are 1.7 and 0.8 days, respectively. Thus, once a pond is infested with *B. calyciflorus*, it will soon crash.

### 2.3. C. kessleri—B. calyciflorus Co-Culture Copper Toxicity Test

**Figure 6:**
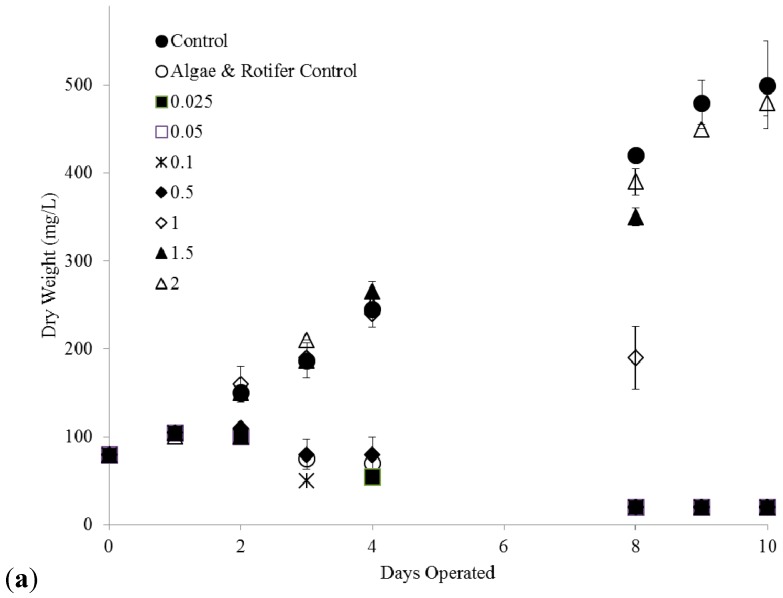

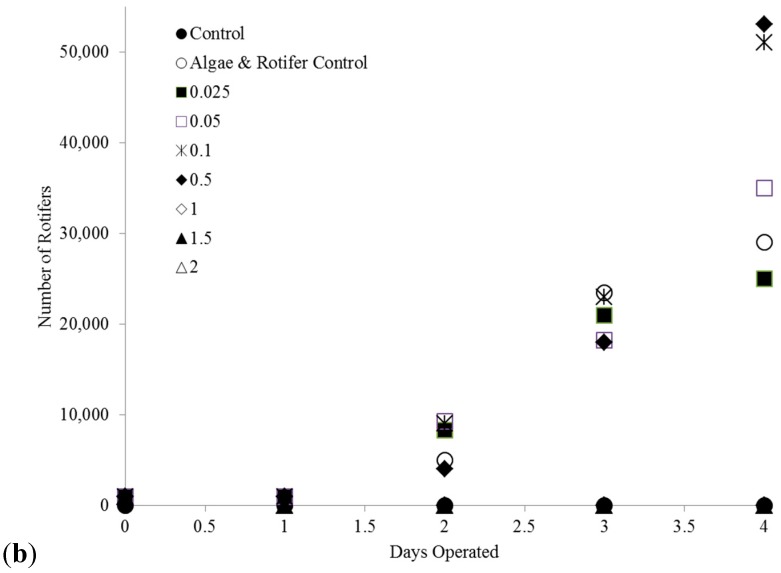
(**a**) *C. kessleri* growth as a function of copper concentration (ppm) at an initial *B. calyciflorus* density of 7 rotifers/mL; (**b**) Increase in *B. calyciflorus* as a function of copper concentration (ppm) while feeding on *C. kessleri*.

Figure 6a,b show the effect of copper on co-cultures of *C. kessleri* and *B. calyciflorus*. After 8 days, only copper concentrations >1.0–1.5 ppm prevented a pond crash by inhibiting the rotifer. Algae growth at copper concentrations >2 ppm did not differ from the controls. Figure 6b shows that 0.5 ppm Cu(II) created the longest lag in rotifer growth (~3 days), yet the highest concentration after 4 days as their food source had more time to grow. In contrast, the lowest Cu(II) concentrations led to the lowest peak rotifer concentrations as the predation rate was the highest in the first few days of the experiment.

The reason why *B. calyciflorus* was able to survive at 0.5 ppm Cu(II), a value ~5× greater than its LC_50_, may be due to the algae taking free copper out of solution; *i.e.*, the rotifers must ingest the algae to experience the full toxic effect of copper in the suspension. In addition, in these tests, using 10-day old cultures, the bound copper concentration increased to 78% which is in agreement with [11] regarding the greater ability of dead algae (*i.e.*, older cultures) to bind copper. It is speculated that as the rotifers consumed, digested, and excreted the algae, the dead algae-material bound the copper, removing it from solution, evidenced by the observation that during a pond crash, the suspension would flocculate and aggregate into a tight pile of biomass on the bottom of the shake flasks.

Once it was determined that copper concentrations greater than 1.5 ppm were needed to prevent a pond crash, fatty acid methyl esters (FAMEs) was quantified in two *C. kessleri* cultures with 2 ppm copper added to determine if copper has any effect on downstream processing. Compared to a control *C. kessleri* culture with no copper added, the FAME percentages were not different. The FAME percentages of the control and copper-added cultures were 19.1 ± 0.5 and 19.6 ± 0.5, (*n* = 9).

Compared to *C. kessleri*, the increased sensitivity of *B. calyciflorus* towards copper may be due to the lower pH of the rotifer stomach. Since rotifers are filter feeders, they indiscriminately ingest algae, free copper, and EDTA-Cu complexes [12]. As the EDTA concentration in the media is 7.9 ppm and the media pH (8.5~9.0) was always higher than the equilibrium pH of the EDTA-Cu complex (8.2), all the copper should be complexed by EDTA or the algae itself. However, once the EDTA-Cu complex enters the low pH of the rotifer stomach, free copper is released and is able to exert its toxic effect. At full-scale, where EDTA may not be added to the media to cut costs, *B. calyciflorus* (and *C. kessleri*) may be more sensitive to copper.

## 4. Experimental Section

This study was conducted in four experimental phases. Phases 1 and 2 were determinations of copper toxicity on *B. calyciflorus* and *C. kessleri* separately. Phase 3 was conducted to study the effect of *B. calyciflorus* on *C. kessleri* growth; *i.e.*, the pond crash rate. In Phase 4, copper was added to a combined culture of *B. calyciflorus* and *C. kessleri* to identify the amount of copper required to control the growth of *B. calyciflorus* while not significantly inhibiting *C. kessleri*. Free and bound copper were also measured in phases 2 and 4 as algae is known to absorb copper. The free copper was measured in the supernatant using Inductively Coupled Plasma Mass Spectrometry and the bound copper was measured using the method by [13]. Last, fatty acid methyl esters (FAME) was measured in the combined cultures to determine if copper has any effect on downstream processing [14].

Thiamine hydrochloride, NaFeEDTA, MnCl_2_·4H_2_O, HCl, NH_4_HCO_3_, and HNO_3_ were purchased from Sigma-Aldrich (St. Louis, MO, USA). NaNO_3_, MgSO_4_·7H_2_O, KH_2_PO_4_, K_2_HPO_4_, NaCl, ZnSO_4_·7H_2_O, CoCl_2_·6H_2_O, CuSO_4_·5H_2_O, and NaMoO_4_·2H_2_O were obtained from Fisher Scientific (Pittsburgh, PA, USA). CaCl_2_·2H_2_O was purchased from AMRESCO (Solon, OH, USA). Crystal Springs^®^ natural spring water was purchased from a local grocery store (Atlanta, GA, USA).

### 4.1. Algae and Rotifer Preparation

*C. kessleri* (UTEX #2228) was cultured in 1 L columns (diameter 5.1 cm and length 64 cm with a tapering bottom end to prevent settling) using modified Bold’s Basal Medium (BBM) under continuous illumination (twelve 40 watt cool white fluorescent light bulbs—4 ft length) and aeration at 25 °C. Modified BBM was prepared according to the method described by Kanz and Bold [15] and was autoclaved at 121 °C for 45 min before use. Every week for about 9 months, the culture was diluted 10× in fresh media to maintain the culture in the exponential growth phase. The optical density (OD) of the culture was monitored at 750 nm using a Spectronic Genesys 20 spectrophotometer (Fisher Scientific, Pittsburgh, PA, USA). The dry weight of the algae was measured according to the procedure explained in [16] and a standard curve was developed relating dry weight to optical density.

*Brachionus calyciflorus* (Gainesville strain) resting eggs were hatched by incubation (Labline Imperial III Incubator) (Barnstead International, Dubuque, IA, USA) at 25 °C in natural spring water under continuous illumination (one 9.5 watt bulb). Neonates hatched after 15–16 h and, at an age of 0–2 h, were either transferred individually using a micropipette. Large numbers of rotifers were counted by sampling 1 mL volumes. This sampling was conducted in triplicate. Once the rotifer density was known, appropriate volumes were transferred to experimental wells to achieve the desired initial rotifer density.

### 4.2. Copper Toxicity and B. calyciflorus

The acute copper LC_50_ toxicity test for *B. calyciflorus* was a 24 h test conducted in both spring water and BBM in 24-well polystyrene plates at 25 °C following [6]. Probit analysis assuming a normal distribution in MS Excel StatPlus (AnalystSoft, Walnut, CA, USA) was used to calculate the rotifer LC_50_. One control and five test concentrations were observed in quadruplicate per plate with 10 neonates in each well. A total of 197 wells were tested to calculate *B. calyciflorus*’*s* LC_50_ in spring water. A copper stock solution (100 mg Cu/L) was prepared using CuSO_4_·5H_2_O and serial dilutions were made in the well plates from 0.002 to 10 ppm Cu(II). Plates were then sealed with parafilm and placed in an incubator in darkness at 25 °C. After 24 h, rotifers were observed under a stereomicroscope (SMZ-2T, Nikon Co., Tokyo, Japan) at 10× magnification. The number of live and dead rotifers was recorded, with rotifers not moving for 10 s regarded as dead. Tests were considered reliable if the mortality of the rotifers in the control did not exceed 10%.

### 4.3. Copper Toxicity and C. kessleri

The effect of copper on algae growth was first tested in 1 L columns and subsequently in 250 mL Erlenmeyer shake flasks. In the shake flasks, the *C. kessleri* copper toxicity tests were adapted from a 72-hour growth inhibition assay [5]. The algae was first grown in 1 L columns and then diluted with BBM to obtain an optical density of 0.1 absorbance units at a volume of 100 mL in the shake flasks. Before each test, glassware was soaked in 10% HNO_3_ for 24 h, washed with deionized water, and autoclaved before conducting each test. The experiments were conducted in quadruplicate on a shaker table (Innova 2100, New Brunswick Scientific, Enfield, CT, USA) at 100 rpm, 25 °C, under continuous illumination using eight 60 W fluorescent light bulbs for 96 h. Test concentrations were 0, 1, 2, 4, 6, and 8 ppm Cu(II) (*n* = 18). The optical density of the algae suspension was measured at 0, 24, 48, 72, and 96 h.

### 4.4. C. kessleri Pond Crash Rate Tests Using B. calyciflorus

*C. kessleri* was cultured as described above except copper was not added and rotifers were added at 1.4, 7, and 14 neonates/mL (*n* = 12). At the same time every day, the optical density was measured and live and dead rotifers were counted by sampling the cultures as described above. This experiment was conducted once.

### 4.5. C. kessleri and B. calyciflorus Co-Culture Copper Toxicity Tests

*C. kessleri* and *B. calyciflorus* were cultured with copper as described above except for the addition of 7 neonates/mL and concentrations of 0.025–2 ppm Cu(II) (*n* = 32). These tests had two controls in quadruplicate: both without copper and one without rotifers (*n* = 8). The optical density of the algae suspension was measured and rotifers were counted at 0, 24, 48, and 72 h. Initial algae OD was 0.2 in all co-culture experiments. Rotifers were counted daily by sampling 1 mL from each 100 mL flask, transferring them to well plates, and observing under the stereomicroscope, then multiplying by 100 to obtain the total number of rotifers in each 100 mL flask. Relative rotifer mortality and algae growth was calculated when the peak rotifer concentration was observed in the positive controls. Relative rotifer mortality was calculated as Abs (1 − (# of rotifers in the experiment/# of rotifers in the positive control)) × 100 (see Equation (1)) with the standard deviation calculated as the average standard deviation of each experimental run/# of rotifers in the positive control × 100 (see Equation (2)).

(1) $$\left|1-\frac{\#\text{ of rotifers in the experiment}}{\#\text{ of rotifers in the positive control}}\right|\times 100$$

(2) $$\frac{\text{average standard deviation of each experimental run}}{\#\text{ of rotifers in the positive control}}\times \;100$$

Relative algae growth is the average growth rate at a Cu(II) concentration/growth rate at the highest Cu(II) concentration × 100 (see Equation (3)) and the standard deviations are the standard deviations of the replicate relative algae growth rates.

(3) $$\frac{\text{average growth rate at a Cu}\left(\text{II}\right)\text{ concentration}}{\text{growth rate at the highest Cu}\left(\text{II}\right)\text{ concentration}}\times 100$$

## 5. Conclusions

This research suggests that copper concentrations above ~0.5 and 5 ppm Cu(II) significantly inhibit *B. calyciflorus* and *C. kessleri* growth. Intermediary concentrations would allow *C. kessleri* to grow while inhibiting *B. calyciflorus*. At the algae pond operating pH of 8–9, a majority of the copper exists as an EDTA-Cu complex which appears to have more of a toxic effect to *B. calyciflorus* than *C. kessleri* due to the low pH of the rotifer stomach. It was shown that copper had no effect on downstream extraction of FAME. These findings give algae farmers a tool to prevent algae pond crashes.
